# Canine Distemper1Read before the Pennsylvania State Veterinary Medical Association, Reading, Pa., October 6, 1896.

**Published:** 1896-11

**Authors:** Howard B. Felton

**Affiliations:** Philadelphia, Pa.


### CANINE DISTEMPER.[1]

BY HOWARD B. FELTON, B.S., V.M.D.,
PHILADELPHIA, PA.

CANINE distemper is a disease that can be traced back to a very early period. Virgil writes of it in his *Georgics*, and there is no doubt that if the records had been properly kept we would find that neolithic man was acquainted with this disorder that gave his dog the running nose and weeping eyes, and was aware that the proper treatment was a supernasal application of the pitchy products of the conifera. We find it described in the hunting-books of the middle ages. Hewitt, an English author, was the first to recognize its contagious nature, which was afterward demonstrated by Karle, Trasbot, Venuta, Krajewski, and Laosson.

Distemper is a disease proper of canines, although it has been observed by Laosson in both domestic and wild cats, and, according to Friedberger and Fröhner, in the fox, wolf, hyena, jackal, and monkey. To this list we must add man, as Zelinski, Nencki, and Karpinski have recently discovered that distemper can be transmitted from the dog to man, causing in the latter tenonitis, or an inflammation of the capsule of Tenon. A full account of this interesting discovery will be found in a translation, by Dr. S. J. J. Harger, of an article on "The Microbe of Canine Distemper," in the *Veterinary Magazine* for June, 1895,

[1] Read before the Pennsylvania State Veterinary Medical Association, Reading, Pa., October 6, 1896.

to which article we acknowledge our indebtedness in speaking of the specific origin of the disease.

Distemper has been claimed by some authors to be variola in the dog. By various others it has been spoken of as typhoid fever, typhus fever, scarlatina. While there are points of resemblance to all these diseases, it has been found, after a careful study and comparison, to be the most closely allied to measles. Both diseases affect young animals; each is ushered in by malaise, anorexia, and chills. In each we have catarrh of the nasal and conjunctival mucous membrane, respiratory troubles, cutaneous eruptions, and lesions of the nervous system. While the resemblance is remarkably close, however, we find in distemper lesions of the digestive tract which differentiate it from measles and assimilate it to typhoid fever, so that taken as a whole it must be regarded as a disease *sui generis.*

As to the specific agent in the causation of distemper much has been done in the line of original investigation and research. Semmer, in 1875, found in the blood and lungs of a dog, which had died of distemper, a micrococcus, and in the blood, lungs, liver, spleen, and kidneys a short, small bacillus. He considered the bacillus to be the specific agent. Laosson continued the researches of Semmer, and cultivated the micrococcus and bacillus from the dog in bouillon, and with these mixed cultures was successful in reproducing the disease. In 1891 Schantyr arrived at the conclusion that the micrococcus heretofore described was only a pyogenic micrococcus. From his opinion he distinguished (1) a disease of young dogs, determined by a small bacillus 1 to 2 $\mu$. in length, grouped, found in the spleen, blood, and the liquid exudates, and liquefying gelatin; their cultures upon serum transmitted the disease; (2) an abdominal typhus caused by a bacillus 0.7 to 2.0 $\mu$. long which did not stain with Gram's method; (3) a typhoid affection due to a bacillus shorter than the preceding, which does not stain with the above method. From 1892 to 1894 Zelinski, Nencki, and Karpinski made a series of observations and discovered in the discharges from the nose and eyes of dogs affected with distemper a micrococcus which was capable of producing in man an inflammation of the capsule of Tenon, and in some cases a diffuse bronchitis and pneumonia. This micrococcus is immobile and measures 8 $\mu$. It can be grown upon bouillon and upon gelatin at 37° C. It is analogous to the streptococcus pyogenes albus, but it decomposes sugar and peptonizes albumin. Since

1893 Galli Valerio, from the Pathological Institute of the Veterinary School of Milan, has made a bacteriological study of this disease, and claims to be the first to have discovered a bacillus in the lungs and central nervous system, inoculations of the cultures from which have produced all the symptoms of distemper. He deduces the following conclusions: 1. In distemper there is found in the lungs and the central nervous system a bacillus whose dimensions vary from 1.25 to 2.5 $\mu$. in length and 0.31 $\mu$. in diameter. 2. This microbe gives characteristic cultures in gelatin at 18° to 20° C. 3. The inoculation of these cultures into the veins, under the skin, and into the lungs of aged dogs does not reproduce the symptoms of distemper. 4. The inoculation of a culture obtained from the brain under the skin of a dog five to six months old has reproduced the disease with its characteristic pulmonary and cerebro-spinal symptoms. It would seem from a review of all these experiments that more than one organism is concerned in the production of this disease, with all its train of complex symptoms.

Distemper usually appears at an early period in the dog's life; in most cases at the time of dentition. It may appear, however, at any age, the extreme limit we have observed being twelve years. The younger the dog the more fatal the disease is supposed to be; but an exception must be made in the case of extremely young dogs, as we have observed in the case of suckling puppies that had contracted the disease from the mother that they almost invariably have it in a mild form and free from complications.

The contagion is extremely volatile and may be communicated by direct contact of the animals with each other, by fomites, by an attendant, or by the air. Semmer relates a remarkable case where the disease was conveyed to two puppies from the carcass of an animal which had been left in the cold for fourteen days prior to an examination.

The period of incubation is from three to five days, shorter in summer than in winter. In cases free from complications this is followed by a slight fever, dulness, and loss of appetite, which last for about four days, the temperature rarely rising above 103° F. At the end of this time the eruption comes out, usually upon the soft parts of the skin and in most cases only in the inguinal region. This eruption may pass through the various stages of erythema, vesicle, pustule, and ulcer, although it is frequently seen simply as an erythema and sometimes only in the form of a vesicle. The eruption does not penetrate the deeper layers of the skin, as we see in variola in the horse and sheep, but is entirely superficial. When the eruption comes out the fever drops, the appetite returns, and the vesicles scale off in about two days. Then comes a secondary eruption which scales off in two days and disappears. There is then a period of convalescence which lasts for about six days, so that the whole attack, if uncomplicated, will last about eighteen days. In highly-bred dogs the first fever may be very high and we may have death occurring due to repercussion, the eruption appearing internally as an intense congestion on the respiratory tract or on the intestinal tract, causing fatal gastro-enteritis. Death may also occur from irritation produced by the pustules. One attack usually conveys immunity, although some dogs will contract the disease a second time, and we have observed one case in which a dog had three separate attacks during a period of four months.

The complications may be divided approximately into three classes: 1. Respiratory. 2. Intestinal. 3. Cerebro-spinal. Respiratory troubles are usually ushered in by a coryza which may disappear in a few days, but in most cases it is followed by congestion of the lungs and broncho-pneumonia, which is indicated by an increase of fever alternating with chills, respirations greatly accelerated, and, in severe cases, flapping of the lips during expiration. On auscultation mucous and sibilant râles can be detected. The animal emaciates rapidly and refuses all food. The discharges from the nose and eyes become muco-purulent. When the nasal discharge becomes mixed with blood it usually indicates the breaking down of a large portion of the lung-tissue and points to a fatal result. The eyes may be affected with a conjunctivitis, which may terminate as such or may be followed by a simple keratitis, which may result in ulcerative keratitis. The intestinal complications may be enumerated as gastritis, gastro-duodenitis, jaundice, and enteritis. Gastritis is indicated by the inability of the stomach to retain food and a persistent vomiting of a frothy mucus which soon becomes mixed with bile. In gastro-duodenitis the matter vomited does not contain bile, but consists of a glairy mucus. Gastro-duodenitis usually ends in enteritis, and frequently, from extension of inflammation of the bile-ducts, in jaundice. Jaundice is almost invariably a fatal complication. We have observed that in distemper enteritis very often assumes a subacute form so far as the manifestation of pain is concerned, although the organs after death may show the signs of intense inflammation. This is no doubt due to the toxic influence of the poison in the blood. There is observed in enteritis first an obstinate constipation, followed by a serous diarrhœa, rapidly becoming dark in color and mixed with blood. The discharges have a peculiar fetor, which is very characteristic. Dysentery frequently sets in, and in a few days the animal becomes greatly emaciated, foul-smelling, and a pitiable-looking object indeed. The discharges are passed frequently in very small quantities, often accompanied by tenesmus, which is liable to cause prolapsus of the rectum. We have found prolapsus of the rectum under these circumstances not to be amenable to treatment. Under the head of cerebro-spinal complications may be mentioned meningitis, myelitis, and chorea. Meningitis usually manifests itself in the form of epileptic fits, in which we have pivoting of the eyes, frothing at the mouth, and champing of the jaws. The animal may throw itself upon its back and utter shrill cries or moans. Meningitis may be exhibited in that rarer form in which the animal walks around continuously in a circle in one direction, either to the right or the left, and appears to be in a sort of stupor, showing indications of brain-pressure. A series of fits in rapid succession may be considered as indicating a fatal result, while one or two, especially if occurring early in the course of the disease, do not have a grave signification. We may look for fits in finely-bred dogs and those of a highly-nervous organization. Myelitis is indicated by a gradual progressing paraplegia, which in most çases is accompanied by anæsthesia of the parts affected. The paralysis usually preceded the fatal result, or, if the animal recovers, there will be in most cases an imperfect use of the hind-limbs. Chorea is a clonic spasm of the voluntary muscles, and may be general or local. It may come on at the end of the period of eruption, or may appear as a sequela some weeks after recovery. The muscular twitching is more marked when the animal is in a state of repose. Associated with it we very often have a high degree of nervous irritability, and the animal cries persistently as if in great pain, sometimes even in his sleep. In other cases the animal remains in good spirits and has an excellent appetite. The general form has been described as a paralysis agitans, and is not amenable to treatment. The constant motion wears the animal out; the muscles atrophy; the animal becomes a miserable skeleton and succumbs to exhaustion. Some local forms, where one set of muscles only or one side of the body is affected, yield to treatment. In others, while the general health may be restored, the muscular twitching persists during life. In rare cases we will observe nervous phenomena simulating locomotor ataxia, in which there is loss of coördination in the movement of the limbs, a staggering, precipitate gait, the legs starting hither and thither in a very peculiar manner and the feet coming down with a stamp at each step. This condition is due to a diseased area affecting the posterior columns of the spinal cord and the posterior nerve-roots. As in the human subject, the use of the eyesight is necessary to prevent the animal from falling. We have not noticed, however, strabismus, ptosis, or neuralgic pains, which are characteristic of this disease in human beings. A rare complication of distemper is lymphadenitis, which we have observed affecting the submaxillary lymphatic glands, the glands on the side of the face, and, in two cases, the thyroid glands, forming abscesses with considerable sloughing of tissue and being very slow to heal. A rare sequela which we have observed and have not seen mentioned before is purpura hemorrhagica, a well-marked case occurring in a spaniel puppy, four months old, one month after recovery from a mild attack of distemper. The alterations are essentially those of a fever, the muscles being of a pale yellow color with little, red ecchymotic spots throughout their substance and the fat turned into little masses of gelatin. The blood is fluid and watery. In enteritis we find ulceration and infiltration of the intestines, especially of Peyer's patches and the solitary glands. In broncho-pneumonia we have congestion of the lungs, bronchi filled with frothy mucus, areas of V-shaped lobular pneumonia, and possibly presence of abscesses in lung-substance. In chorea and myelitis we have infiltration of round lymphoid cells into the spinal cord, medulla oblongata and cerebellum, and granular degeneration of the nerve-cells.

The treatment is essentially symptomatic. Many cases require no treatment but good hygienic surroundings and careful feeding. There is no disease where careful nursing is more urgently required. The eyes need frequent cleansing to prevent ulceration. The bowels need to be carefully looked after to prevent enteritis. The animal must be kept from drafts to prevent pneumonia, and, if a nervous subject, must be treated very gently to avoid fits. While these complications may occur despite the best of care, yet the possibility of preventing them should cause us not to relax our vigilance. For conjunctivitis we bathe the eyes frequently with hot water and apply a solution of boric acid, 10 grains to the ounce. For a simple keratitis we use, thrice daily, a collyrium containing ¼ grain atropine and 2 grains sulphate of zinc to the ounce. We have never found it necessary to use a stronger solution than this. When ulceration of the cornea sets in we use, in addition to the collyrium above mentioned, dry oxide of zinc dusted upon the ulcers, being sure to have the powder free from grit and ground very fine. Our method is first to use the collyrium and then the powder. In some cases we have had good results from the use of honey in addition to the other remedies, applying two drops in the eye morning and evening. When the cornea has ruptured and the aqueous humor run out, granulations frequently spring out from the seat of the ulcer, which have been called staphyloma. It is often necessary to remove these with the knife, as they are exquisitely tender and cause the animal great pain and discomfort. Shoemaker's wax upon the nose is not to be despised, but we have found better results from fumigations of tar, which seem to exercise a decidedly beneficial action upon the disease, especially upon the bronchial complications. Care must be taken, however, not to make the fumes too dense, as in this case they prove too irritating. We find quinine to be indispensable in the treatment of distemper. In pneumonia we combine it with muriate of ammonium, giving from 1 to 3 grains of each in capsules, according to the size of the dog, three or four times daily. We also employ it in the intestinal complications combined with subnitrate of bismuth. We have had good results in pneumonia, especially in cold weather, from the use of a jacket stuffed with cotton-batting sewed around the chest and neck, placed on early in the attack and left on until some time after convalescence. In bad cases of pneumonia we have used $\frac{1}{120}$ to $\frac{1}{80}$ of a grain of strychnine as a respiratory stimulant with good results. We also give milk-punches frequently in small amounts, alternating with beef-tea when the animal grows tired of them. In gastritis and in mild forms of diarrhœa we have found salol and subnitrate of bismuth to be a good combination. In cases of persistent vomiting we give half-drop doses of carbolic acid and iodine every hour, together with small doses of egg-albumin given frequently, alternated with milk and lime-water. In bad cases of diarrhœa, when the discharges are tinged with blood, and in dysentery we employ a combination of opium 1 grain, sulphocarbolate of zinc 1 grain, and bismuth subnitrate 5 grains. Also enemata of starch-water. We have found the best diet in dysentery to be raw meat or meat slightly broiled in butter, cut up finely, and given in small quantities. This is completely digested in the stomach before it reaches the inflamed bowel. We do not use purgatives, but employ olive oil for its laxative, emollient, and nutritive qualities. For epileptic fits we employ the hot bath and give mixed doses of pot. bromide and chloral, 5 grains of each, repeating every three hours and increasing the dose if necessary. Where it is necessary to use this remedy for some time we substitute bromide of sodium for the potassium salt, as it can be given in larger doses and is better tolerated by the stomach. In cases of locomotor ataxia when the animal travels around in a circle we have never had any good results from treatment. It would seem in the latter case that trephining is indicated if the proper spot could be found. In chorea we have tried every remedy proposed thus far, and must say that the only good results we have ever obtained have been by the employment of arsenic. We have not obtained the results which M. Monfallet gets by the use of quinine. It may be, however, that the cases which he cured did not have chorea as a result of distemper. It is our opinion, and that of a number of physicians with whom we have conversed, that chorea following distemper in the dog is of a much more intractable kind than that seen in children. We believe it to result largely from the anæmic condition of the blood, and the granular degeneration of the nerve-cells in this disease indicates that it is a very serious lesion indeed. In arsenic we have the valuable properties of a blood-tonic and a special stimulant to the nervous system. We begin with two drops of Fowler's solution thrice daily, increasing one drop every third day and pushing it up almost to the verge of poisoning, stopping altogether when the animal begins to vomit, and after two days commencing again with the smallest dose. In some dogs we find an idiosyncrasy against the use of Fowler's solution due to the lavender in its composition, and in these cases we employ the liquor acidum arseniosum, the dose being the same. With this treatment we have cured a fair proportion of cases, but, alas, far too few. The great trouble in the treatment of chorea is in the length of time it takes to effect a cure, the owner usually becoming disheartened at the slow rate of improvement and ordering the animal destroyed. Many cases will take from three to six months to effect a cure. In others the twitchings may persist for a year and then disappear. We are anxiously looking for the discovery of an antitoxin which shall be both preventive and curative, and be a much more powerful weapon than any we are able to employ at present in combating this disease.

